# Novel Lead Halide Perovskite and Copper Iodide Materials for Fluorescence Sensing of Oxygen

**DOI:** 10.3390/bios15030132

**Published:** 2025-02-21

**Authors:** Jingwen Jin, Yaning Huang, Chen Zhang, Li Zhang, Shaoxing Jiang, Xi Chen

**Affiliations:** 1 Institute of Analytical Technology and Smart Instruments, College of Environment and Public Health, Xiamen Huaxia University, Xiamen 361024, China; jinjw@hxxy.edu.cn (J.J.); zhangc@hxxy.edu.cn (C.Z.); zhangl@hxxy.edu.cn (L.Z.); 2 Information Center, Xiamen Huaxia University, Xiamen 361024, China; hyn@hxxy.edu.cn (Y.H.); jiangs@hxxy.edu.cn (S.J.); 3 Department of Chemistry, College of Chemistry and Chemical Engineering, Xiamen University, Xiamen 361005, China

**Keywords:** oxygen sensing, fluorescence, doping, lead halide perovskite, copper–iodine clusters, copper-doped nanocrystals

## Abstract

The most commonly used optical oxygen sensing materials are phosphorescent molecules and functionalized nanocrystals. Many exploration studies on oxygen sensing have been carried out using the fluorescence or phosphorescence of semiconductor nanomaterials. Lead halide perovskite nanocrystals, a new type of ionic semiconductor, have excellent optical properties, making them suitable for use in optoelectronic devices. They also show promising applications in analytical sensing and biological imaging, especially manganese-doped perovskite nanocrystals for optical oxygen sensing. As a class of materials with diverse sources, copper iodide cluster semiconductors have rich structural and excellent luminescent properties, and have attracted attention in recent years. These materials have adjustable optical properties and sensitive stimulus response properties, showing great potential for optical sensing applications. This review paper provides a brief introduction to traditional oxygen sensing using organic molecules and introduces research on oxygen sensing using novel luminescent semiconductor materials, perovskite metal halides and copper iodide hybrid materials in recent years. It focuses on the mechanism and application of these materials for oxygen sensing and evaluates the future development direction of these materials for oxygen sensing.

## 1. Introduction

Oxygen is one of the molecules most closely related to human life. The oxygen content in the atmosphere and water reflects the environmental quality and the ecosystem. The oxygen content in blood and cells is an important parameter for physiological health and clinical medicine. Moreover, oxygen not only widely participates in industrial catalysis, crop cultivation, and biological fermentation processes but also affects mining safety and food storage. For example, in food preservation and packaging, the global food processing industry is worth more than USD 4 trillion annually. In addition, the global wastewater treatment market was USD 55 billion in 2020. The hydrogen fuel cell market is expected to reach USD 42 billion by 2030. Therefore, since the Lavoisier era, the development of efficient oxygen sensing methods has been of great significance in fields such as environmental science, biomedicine, chemical production, and the food industry. The early adopted Winkler redox titration method yields accurate results, but it has problems of cumbersome operation and inability to continuously monitor changes in oxygen content. Subsequently, the Clark oxygen electrode method has been vigorously developed due to its fast and real-time sensing characteristics. However, electrochemical sensing methods based on this method require continuous consumption of oxygen and regular replacement of electrolytes, affecting the accuracy of the sensing results. In recent years, optical sensing methods have attracted widespread attention due to their advantages such as rapid response, high sensitivity, recyclable detection, and anti-electromagnetic field interference, and have developed into important oxygen sensing methods [1].

Molecular oxygen is a typical quencher of fluorescence (FL) and phosphorescence. The vast majority of optical oxygen sensing methods are realized based on the principle of FL dynamic quenching including gaseous oxygen and dissolved oxygen [2,3,4]. Therefore, when selecting oxygen sensing probes, in addition to meeting the requirements of having good photobleaching resistance and an appropriate emission wavelength, the sensing material should also possess a longer FL lifetime and a larger specific surface area to enhance the collision probability between the luminescent material and molecular oxygen and maximize the optical quenching ability of oxygen molecules. Most optical oxygen sensors are based on the dynamic quenching between triplet oxygen molecules and the excited state of the sensing material. Since this process is a photophysical rather than a photochemical process, it is completely reversible. In dynamic quenching, the absorption behavior of the material is not affected, while the FL behavior shows a decrease in FL intensity and decay time. The relationship between FL intensity or decay time and oxygen concentration ([O_2_]) can be reflected by the Stern–Volmer equation, and its simplest form is shown as Equation (1):

(1) $$\frac{I_{0}}{I}=\frac{\tau_{0}}{\tau }=1+K_{sv}\left[O_{2}\right]$$

where I and τ refer to the luminous intensity and decay time, respectively, I_0_ and I are the luminous intensity of the sensor under aerobic and anaerobic conditions, respectively, τ_0_ and τ are the luminous lifetime of the sensor under aerobic and anaerobic conditions, respectively, Ksv is the Stern–Volmer constant and [O_2_] is the concentration of oxygen in the sample.

The core of the optical sensing method is the selection and design of oxygen sensing materials. Polycyclic aromatic hydrocarbons (PAHs) represented by pyrene and its derivatives are the earliest used optical oxygen sensing materials. Metal complex oxygen-sensitive probes can satisfy the above requirements. As metal complexes usually have a strong FL intensity and a long FL lifetime, they can exhibit high oxygen sensing sensitivity, and include complexes such as transition metal–organic complexes [5,6,7,8,9,10] and metal porphyrin complexes [11,12,13]. Through theoretical calculations, it was found that the highest occupied molecular orbital (HOMO) of metal–organic complexes is typically composed of the metal component, and the lowest unoccupied molecular orbital (LUMO) is mainly composed of the organic ligand part. Therefore, their electronic transitions have the property of metal–ligand charge transfer. In other words, the luminescent properties of the corresponding metal–organic complexes can be modulated by the ligand, thereby achieving the regulation of oxygen sensing sensitivity. Huang et al. [14] designed a series of iridium complexes. By adjusting the internal conversion efficiency between the two excited states of the complexes, the dual phosphorescence emission characteristics and the response to oxygen were rationally adjusted, constructing an oxygen-sensitive molecular probe based on the ratio sensing method and expanding the sensing range from hypoxia to hyperoxia. The core part of the optical sensing method is the screening and design of oxygen sensing materials. Polycyclic aromatic hydrocarbon compounds represented by pyrene and its derivatives are the earliest types of optical oxygen sensing materials used.

At present, the most extensively investigated oxygen sensing materials are represented by long-lived noble metal complexes such as ruthenium polypyridyl complexes and platinum porphyrin complexes [15]. Some of these materials have reached the level of device application in terms of oxygen sensing sensitivity. O_2_ molecules quench the FL of the above-mentioned materials through non-radiative transfer of the excited state energy, which is currently the most representative oxygen sensing mechanism. Although the FL intensity or lifetime of these materials is highly sensitive to the variation in oxygen content, the Stokes shift in PAHs is limited and they are prone to photobleaching. Moreover, noble metal complexes also have issues such as complex synthesis and high cost. To optimize the performance of oxygen sensing materials and deepen the research on oxygen sensing mechanisms, researchers have successively developed various novel luminescent materials in recent years, achieving effective sensing of O_2_ molecules, among which oxygen-sensitive semiconductor luminescent materials are included. It is known from the electron spin state of the O_2_ molecules in the highest occupied molecular orbital that the ground state O_2_ (Σ_g_^−^) has unpaired electrons on each of the two anti-bonding π orbitals (π_x_* and π_y_*) with the same energy, thus exhibiting strong electron-withdrawing properties and high reactivity. Previous studies have discovered that O_2_ can influence the photoexcitation and deexcitation behaviors of semiconductor luminescent materials through multiple electron-involving pathways, thereby causing reversible quenching or enhancement of the PL of some II-VI semiconductor nanocrystals (NCs) [16,17]. This provides an alternative idea for the research of optical oxygen sensing from a focused perspective and promotes the development of oxygen-sensitive semiconductor luminescent materials.

## 2. Oxygen Sensing of Luminous Organic Molecules

### 2.1. Oxygen Sensing System for Polycyclic Aromatic Hydrocarbons (PAHs)

**Figure 1:**
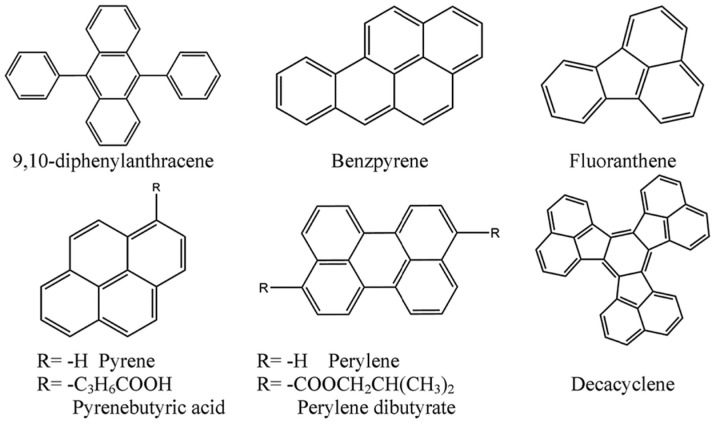
Chemical structures of common PAH-based oxygen-sensitive probes (OSPs) [1].

In PAHs, except for some containing nitro or carbonyl structures, almost all PAHs have strong and stable PL characteristics. These molecules have a PL lifetime of up to 200 ns, which is easily quenched by oxygen. In particular, pyrene and its derivatives have become the first generation of oxygen photochemical sensing compounds. At ambient temperature, they also show good oxygen sensitivity and low temperature absorption coefficients. However, due to the lack of good solubility in polymers, oxygen sensors based on pyrene are prone to the problem of luminescent molecule aggregation [18]. To overcome these shortcomings, functional side chains can be chemically modified for pyrene derivatives [19]. Pyranobutyric acid is a pyrene derivative with good water solubility, with its maximum absorption peak at 355 nm and maximum monomer emission peak at 396 nm, and a PL lifetime of 200 ns. This material can be fixed on glass by DMF solution treatment and used for oxygen sensing. Using similar strategies, researchers have developed quenching-type oxygen sensors made from a combination of various PAHs and polymers, with the 1-pyridinebutanoic acid sensor showing the best performance parameters such as sensitivity, response time, and reproducibility. The chemical structures of some common oxygen-sensitive PAHs are shown in Figure 1. Due to the significant influence of humidity on the oxygen sensing results of PAHs, these materials are suitable for sensing oxygen in non-water samples.

### 2.2. Oxygen Sensing Using Room-Temperature Phosphor Organic Molecules

**Figure 2:**
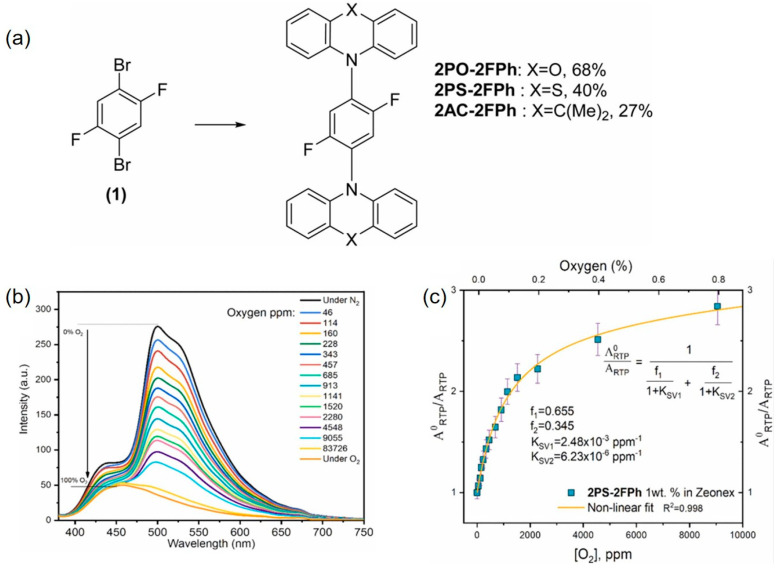
(**a**) Synthesis of 2,5-disubstituted-1,4-phenylene derivatives; (**b**) RTP spectra of 2PS-2FPh; (**c**) Stern-Volmer plots for 1% solid solution of 2PS-2FPh in Zeonex recorded at the different concentrations of oxygen in the atmosphere of the sample [26].

Materials with room-temperature phosphorescence (RTP) properties have attracted widespread attention due to their potential applications in chemical and biological sensors, security protection, and photo-dynamic therapy based on biological imaging [20,21]. Among the materials with RTP properties, organic RTP emitters without metal have the advantages of simple synthesis and high cost-effectiveness. To achieve efficient RTP in organic compounds without metal, two strategies are usually employed. One method is to introduce halogen atoms (Br or I) or heteroatoms (N, S, O) to create a heavy atom effect or (n → π*) transition structure, which can enhance spin–orbit coupling (SOC) to achieve efficient intersystem crossing (ISC) [22,23]. Another method is to use rigid environments to suppress the non-radiative deactivation process of molecules, such as crystallizing the material, adding hydrogen bonds and halogen bonds, or using host matrices [24,25]. Skhirtladze et al. [26] constructed a series of optical oxygen sensors using different electron donor groups (phenothiazine, phenoxazine, and acridine) and the electron acceptor group of 1,4-difluorobenzene (Figure 2). The research results show that when one of the molecules of 2PS-2FPh is doped into a rigid medium Zeonex, a material can be obtained that generates strong RTP in air. Molecular dynamics simulations indicate that the medium’s disordered conformation and rigidity can significantly broaden the single-excited-state-triplet splitting of 2PS-2FPh molecules, making the charge-transfer triplet (^3^HLCT) more localized, thereby promoting 2PS-2FPh’s RTP and high oxygen sensitivity.

Metal-free RTP organic molecules with oxygen responsiveness are beginning to attract researchers’ interest. However, these materials typically exhibit intense phosphorescence only in the crystalline phase [27] and do not have the same properties in solid films. So far, there are few reports of the use of these materials in the determination of dissolved oxygen content. Therefore, to enable these materials to play a role in a wider range of application scenarios, oxygen sensing research based on such materials still has a long way to go.

## 3. Oxygen Sensing of Luminous Metal Complexes

### 3.1. Oxygen Sensing of Transition Metal Complexes

**Figure 3:**
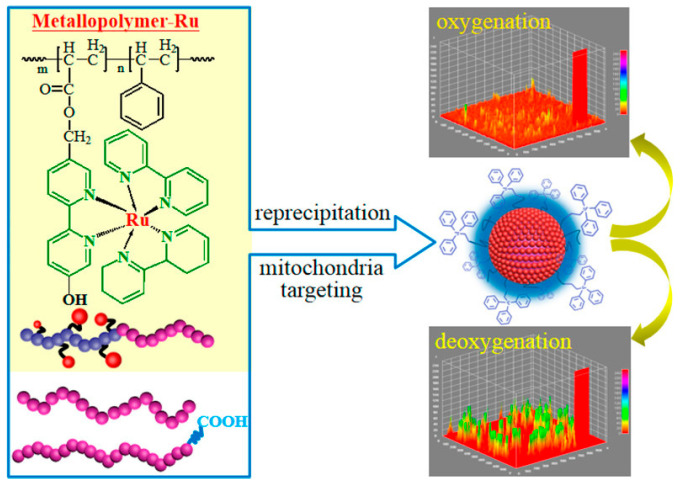
Schematic diagram of nano-oxygen sensor based on Ru-containing metallopolymer [31].

The luminescent transition metal complexes (TMCs) not only can exhibit relatively long lifetimes and strong visible light absorption, but also have stronger light stability than the organic molecules. The spectral and chemical properties of these complexes can be adjusted to some extent by modifying the coordination complex structure. Under light excitation, TMCs usually undergo a metal–ligand charge transfer process to form excited triplet or even higher multiplicity states. The long-lived excited states provide sufficient energy exchange time for the material to interact with oxygen molecules, resulting in high sensitivity to oxygen. In addition, the energy of the triplet state is lower than that of the singlet state, so the phosphorescence wavelength is longer than the FL wavelength. The large Stokes shift can effectively reduce the spectral background. Early oxygen sensing research based on Ru(II) complexes was mainly conducted using commercially available reagents prepared by following a simple protocol, such as [Ru(bpy)_3_]^2+^ (where bpy = 2,2′-bipyridine) [28] and [Ru(phen)_3_]^2+^ (where phen = 1,10-phenanthroline) [29]. Owing to good light stability and oxygen response performance, Ru(II) complexes have been widely used in the field of oxygen sensing. In addition to Ru(II) complexes, some transition metal complexes based on different central metal ions, such as Ir(III), Os(II), and Re(II), have also shown long PL lifetimes and high PL efficiencies, thus also possessing potential for oxygen sensing applications [6,30]. However, the quantum yield and sensing performance of these luminescent oxygen probes are usually affected to some extent by the diffusion barrier or aggregation-induced quenching (ACQ) effect of the matrix [25]. To address this issue, as shown in Figure 3, Peng’s research team proposed a strategy for a luminescent nanoprobe based on a Ru(II) metal polymer [31], where the oxygen probe [Ru(bpy)_3_]^2+^ is appropriately grafted onto the surface of the polymer to allow the probe molecules to be dispersed well with each other. The results show that the nanoprobe exhibits strong red FL without ACQ effects and is highly responsive to oxygen.

**Figure 4:**
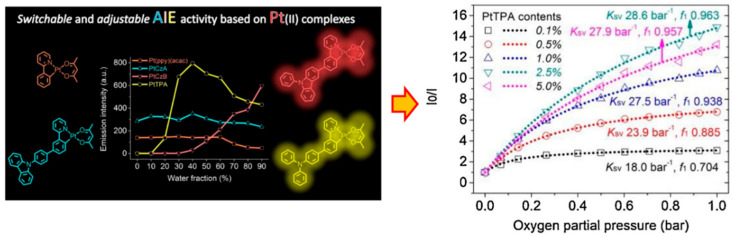
Oxygen sensing study based on Pt(II) complexes with adjustable AIE activity [36].

More effective methods of weakening and even overcoming ACQ come from the ground-breaking research on thiolato derivatives by Tang et al. in 2001, namely the aggregation-induced emission effect (AIE) [32]. The sensitivity of Pt(II) complexes to external molecular oxygen means they are widely used in the manufacture of functional materials and sensors [33,34]. Among them, cyclic metal-free Pt(II) complexes as PLmaterials have shown significant superiority in excitation state lifetime and resistance to photobleaching [35]. As shown in Figure 4, Yang et al. synthesized three AIE Pt(II) complexes, PtCzA, PtCzB, and PtTPA, with similar substituent structures, and compared their PL behaviors through a series of characterizations [36]. Oxygen sensing tests showed that the controllable AIE property of Pt(II) complexes can effectively improve the uniformity of the PL oxygen sensor, thereby achieving better sensing performance. However, there have been only a few reports on Pt(II) complexes with AIE activity to date. Moreover, most oxygen sensing materials based on TMCs only exhibit PL AIE properties [37], while the number of reports on PL AIE properties of such materials is still very small [38,39]. Therefore, more research is needed to fully exploit the optical sensing potential of these materials.

### 3.2. Oxygen Sensing of Metalloporphyrin Compounds

**Figure 5:**
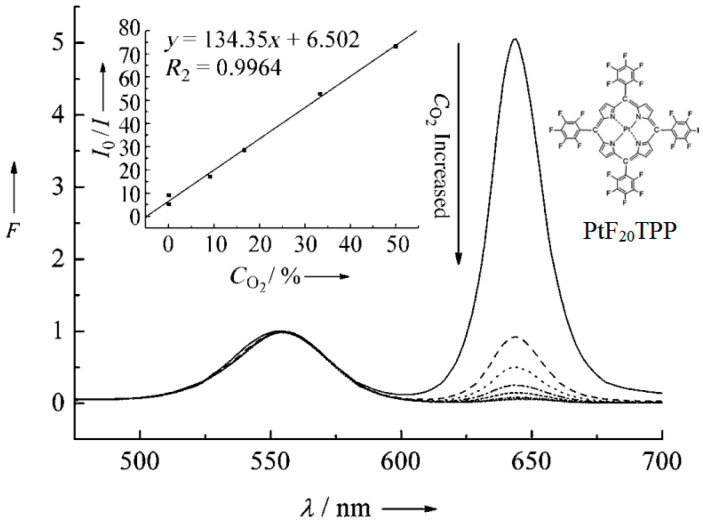
FL spectrum of [meso-tetrakis(pentafluorophenyl)-porphyrinato]platinum(II) (PtF_20_TPP) and its sensing responses towards various concentrations of oxygen [41].

Although the TMCs mentioned above have been used to construct oxygen sensing systems, the number and type of ligands that the central metal can accommodate are limited. Metalloporphyrin (MPP)-type complexes are another type of oxygen sensing material that has been developed in the past few decades. Because they have a variety of peripheral groups, MPPs can be easily functionalized through modification. The most common MPPs are Pd(II) and Pt(II) porphyrins, which usually have strong absorption in the range of 390 to 420 nm and weak absorption above 500 nm, with a sharp and intense red emission, with a Stokes shift of up to 250 nm. Compared to TMC sensing materials such as Ru(II), MPP-type complexes have higher room temperature PL quantum yields and longer lifetimes, and have superior optical properties for oxygen sensing. Based on these advantages, MPP-type complexes have become the most common oxygen sensing materials [40]. Recent research on using MPPs for oxygen sensing has utilized their thermally activated delayed fluorescence (TADF) properties. Chen et al. [41] developed a novel colorimetric oxygen sensor using [meso-tetrakis(pentafluorophenyl)- porphyrinato] platinum(II) (PtF_20_TPP) and cadmium telluride (CdTe) quantum dots (QDs), by which oxygen concentration could be defected with precise, distinct, and unparalleled color observation. In this sensing approach, as shown in Figure 5, the Pt complex, PtF_20_TPP, gave a red FL emission at the wavelength of 650 nm, which could be quenched by different concentrations of oxygen.

**Figure 6:**
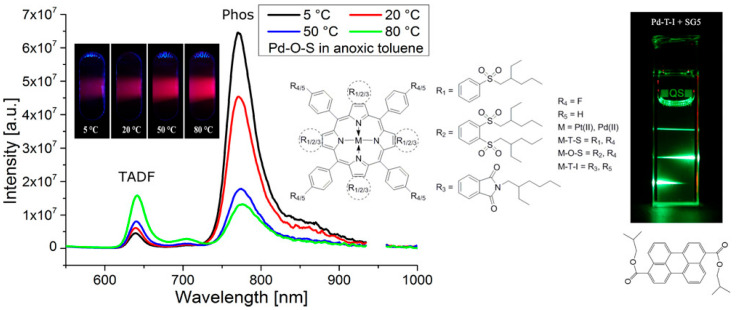
Oxygen and temperature dual sensing based on platinum (II) and palladium (II) benzoporphyrins containing alkylsulfone groups [42].

Zach et al. [42] achieved the first dual sensing of oxygen and temperature using a single emitter. As shown in Figure 6, the team used a four-step synthetic method using a template to prepare highly electron-deficient Pt(II) and Pd(II) benzoporphyrin complexes containing alkylsulfone groups. In solution and polymer matrices, these complexes can produce highly efficient TADF at high temperatures due to their small singlet-triplet energy gap, which meets the requirements for optical oxygen sensing. In addition, their emission intensity is proportional to the temperature, so that dual sensing of oxygen and temperature can be based on a single emitter. In dual sensing, temperature information is obtained from the TADF-to-phosphorescence ratio, and oxygen concentration is determined by phosphorescence or TADF decay time.

In the research and application of oxygen sensing using molecular luminescent compounds, apart from the early-used PAHs and RTP compounds, TMCs with Ru or Pt as the central atom [43,44] have been widely applied. Indeed, some bioluminescent compounds, including D-luciferine, celenterosine, flavin mononucleotide, etc., have also been the focus of studies on the mechanism of oxygen sensing and biological imaging [45,46], and related research and application reviews have been published [47].

## 4. Oxygen Sensing of Luminescent Semiconductor Materials

### Oxygen Sensing of Perovskite-Doped Materials

A variety of luminescent materials have already demonstrated their potential in oxygen sensing applications. However, to meet the increasingly widespread demand for oxygen sensing, some key issues still need to be addressed. Although organic FL molecules have the longest history in oxygen sensing, their poor light stability and small Stokes shift limit their wider application. In addition, moisture stability is also an issue that these materials need to address. Metal–organic complexes, especially MPPs, have fully overcome these disadvantages and have longer FL lifetimes, thus becoming the most mainstream sensing materials. However, the universal disadvantage of MPPs and other metal complexes is their toxicity, which is very unsuitable for their applications in biology and medicine. On the other hand, these materials need complicated synthesis steps, which leads to the high cost of the sensors. In addition, not all MPPs have good light stability, which is an important factor to consider in long-term measurements. In the past three decades, researchers have developed various types of luminescent semiconductor materials, including quantum dots, luminescent noble metal nanoparticles, and upconversion emitters. Luminescent semiconductors have good light stability, adjustable optical bandgaps, high PL quantum yield, etc. The length of the FL lifetime is one of the major factors affecting the sensitivity of oxygen sensing materials. Quantum dots of II-VI and III-V semiconductors, such as those represented by quantum dots, have wide excitation spectra, narrow emission spectra that can be adjusted, high quantum yields, and good resistance to photobleaching, etc. They have received extensive and continuous attention in the past two decades due to their superior PL characteristics [48]. In 1998, Jr et al. [49] and Nie et al. [50] opened up the research field of fluorescent labeling with semiconductor NCs. Since then, the optical properties of semiconductor NCs have been fully utilized in the fields of analytical sensing and cell imaging [51,52,53].

Porous and uniform channels can ensure sufficient contact between oxygen and the sensing material, making it effective for oxygen content determination. Dispersing the sensing material in a polymer matrix [1,54] or a porous material [55] is a common method, which can make the sensing material evenly distributed while providing space for oxygen to freely move. Additionally, for block-shaped materials, the stacking pattern of the crystal lattice can result in different void fractions, leading to different oxygen quenching effects. McGee et al. [56] designed a luminescent ruthenium complex crystal [Ru(phen)_3_(tfpb)_2_] with a pore structure; as oxygen can freely pass through the crystal’s pores, it can serve as an effective oxygen sensing crystal material. Subsequently, the research group found that ruthenium complexes with the same molecular formula but different crystal packing patterns [Ru(phen)_3_(PF_6_)_2_] exhibited different oxygen sensitivities [57], with the oxygen quenching value ((*I*_nitrogen_ − *I*_air)_/*I*_nitrogen_) reaching 0.36 and 0.33 for the chiral structure crystal, while only 0.05 for the achiral structure crystal, due to the larger voids in the chiral structure crystal with a non-close-packed structure, and the open pores allowing oxygen molecules to diffuse freely within the crystal. Due to the high cost of ruthenium complexes, the research group’s subsequent work also designed one-valent copper complex oxygen-sensitive probes with different void fractions, which also had excellent oxygen sensing performance [58].

Lead halide perovskites (LHPs) are an emerging semiconductor luminescent material that not only has diverse synthetic methods and low material costs, but also has a unique electronic and band structure that endows it with superior optical properties, including high light emission quantum yield, high defect tolerance, easily adjustable emission spectrum, etc. It has been widely studied in the field of optoelectronic devices. In addition, compared to traditional inorganic semiconductor luminescent materials, the soft ionic lattice of LHPs makes its PL more sensitive to specific factors in the environment. Therefore, by tuning the composition and structure of LHPs, not only can a variety of optical properties be generated, but also the optical sensing ability can be explored and new sensing mechanisms can be proposed, which is of great significance for expanding the application of this material in analytical sensing. There have been reports in the literature of diverse oxygen-sensitive luminescent behaviors in LHPs. Based on the electron spin state of the O_2_ molecule in the highest occupied molecular orbital, it can be known that the ground state O_2_(^3^Σ_g_^−^) has two unpaired electrons in the anti-bonding π orbitals (π_x_* and π_y_*) with equal energy, so it has strong electron-withdrawing ability and high reactivity. Previous studies have found that O_2_ can affect the photoexcitation and deexcitation behavior of semiconductor light-emitting materials through various electron-involving pathways, resulting in reversible quenching or enhancement of the PL of some II-VI semiconductor NCs [16,17]. This provides another line of thought from a theoretical perspective for optical oxygen sensing research and drives the development of oxygen-sensitive semiconductor light-emitting materials. By analyzing different morphologies of CsPbBr_3_ [59,60], researchers have discovered that O_2_ molecules can interact with CsPbBr_3_ nanocubes through collisions to extract the photogenerated electrons, leading to the dynamic quenching of the LHPs PL. On the other hand, O_2_ can also passivate the surface hole traps of CsPbBr_3_ nanowires, nanoflakes, and single crystals, enhancing the LHPs PL without altering the exciton recombination dynamics. The oxidation of the MAPbI_3_ interstitial iodine by O_2_ eliminates deep-level defects, resulting in an enhancement of the HLP PL [61,62]. Oxygen quenching of the MAPbI_3_ emission by O_2_-assisted photoinduced etching also causes a blue shift in the emission wavelength, ultimately leading to complete quenching of the emission [63]. Zhu et al. [64] utilized cadmium telluride quantum dots encapsulated with cysteine for oxygen sensing applications, but the degree of oxygen quenching of the FL in this work was relatively weak, and the problems of surface oxidation and light flashing that lasted several seconds were not effectively addressed. These issues indicate that the application of luminescent semiconductor materials for oxygen sensing still has a long way to go. With the progress of wet chemical synthesis in recent years, more and more new semiconductor materials have been paid attention to and one of the most notable examples is the doping material of lead halide perovskite.

**Figure 7:**
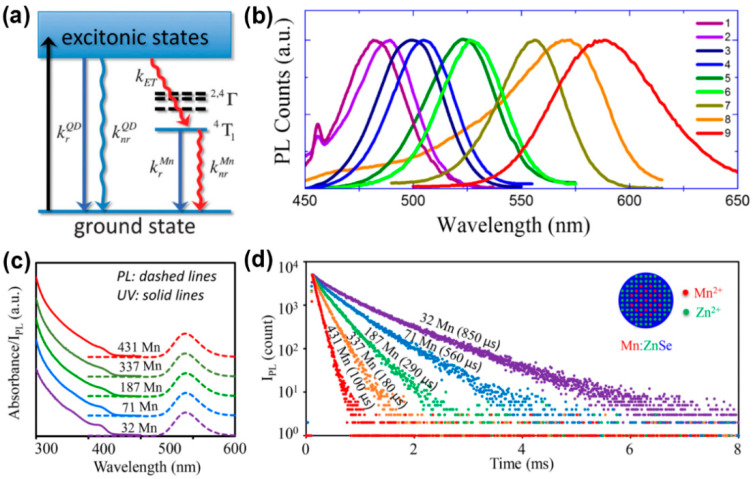
(**a**) The exciton recombination process of Mn^2+^-doped QDs [65]; (**b**) Mn emission band from different Mn:ZnSe/CdSe/ZnSe QDs [69]; (**c**) absorption and PL spectra of Mn:ZnSe NCs with different amounts of Mn^2+^ ions; (**d**) PL decay curves of Mn emission [70].

Appropriate doping of metal ions in semiconductor NCs can endow NCs with novel optical, electrical and magnetic properties without changing the crystal structure. Among them, the FL of semiconductor NCs doped with Mn^2+^ has a long FL lifetime, large Stokes shift and paramagnetic properties, attracting people’s interest. One situation where semiconductor NCs generate doped FL is when impurity ions form impurity levels within the bandgap of the host NCs, resulting in doped FL via exciton energy transfer, which mainly reflects the properties of the impurity ion itself, such as Mn doping. As shown in Figure 7a, the 3d^5^ configuration of Mn^2+^ is forbidden, but when it is doped into suitable semiconductor NCs, the d electron level accepts part of the exciton energy, activating the ^4^T_1_→^6^A_1_ spin relaxation, thus producing a specific Mn emission peak at around 585 nm, with a large Stokes shift and a millisecond-level long PL lifetime [65,66]. In addition, there is also a theory that Mn^2+^ captures the photogenerated hole and then combines with the electron to form the excited state of (Mn^2+^)*, and then releases the energy in the form of light [67]. Although the Mn-doped FL is determined by ^4^T_1_→^6^A_1_, the degree of splitting of the d electron level of Mn^2+^ is greatly affected by the change in its crystal field environment, and there may be multiple FL levels at the same time, so there will be a spectral broadening (peak width at half height of 50–70 nm).

**Figure 8:**
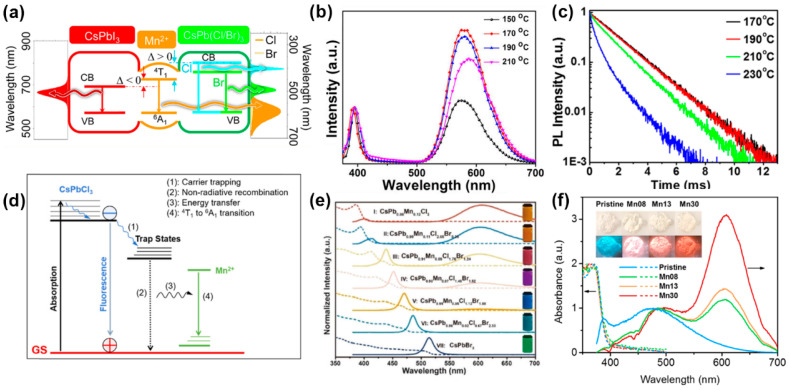
(**a**) Energy level diagram of Mn:CsPbX_3_ NCs; (**b**) FL emission spectra of CsPb_x_Mn_1−x_Cl_3_ NCs prepared at different temperatures; (**c**) Mn emission decay curves of the Mn:CsPbCl_3_ NCs synthesized at different temperatures [74]; (**d**) schematic energy levels and electronic transitions in Mn-doped CsPbCl_3_ NCs [75]; (**e**) absorption and FL emission spectra of Cs(Pb_x_Mn_1−x_)(ClyBr_1−y_)_3_ NCs; (**f**) absorption and FL emission spectra of Mn-doped 1D lead bromide perovskites.

The degree of overlap between the wave function of the exciton and the wave function of the d electron determines the strength of the exchange coupling, which in turn depends on the chemical and structural details of the d-dots. The chemical environment of Mn^2+^ in the d-dots directly affects the wavelength and FL lifetime of the doped FL. Dohner et al. [68] controlled the size of CdS NCs to change the wavelength of the Mn emission peak by 40 nm in the red-yellow light region, and proposed that when the impurity is distributed in the surface region of the NCs, the impurity is in a distorted tetrahedral coordination environment, which will result in energy reduction, so the wavelength of the Mn emission peak redshifts. Hazarika et al. [69] designed and synthesized multi-layer core/shell structured Mn:ZnSe/CdSe/ZnSe QDs. By adjusting the thickness of the CdSe layer and the doping position of Mn^2+^, the strain of the NCs was controlled. When Mn^2+^ was farther away from the CdSe/ZnSe interface, the wavelength of the Mn emission peak gradually red-shifted from 480 nm to 580 nm, successfully covering the visible light region (Figure 8b). The FL decay kinetics of Mn impurities in d-dots are different due to their different chemical environments. The FL lifetime is related to the spatial distribution and concentration of impurities. Pu et al. [70] prepared Mn:ZnSe NCs by the co-nucleation method and found that when the particle size remained unchanged, by reducing the number of Mn^2+^ in each NCs from 431 to 32, the FL lifetime of the doped FL increased from 100 μs to 850 μs. Meanwhile, the chemical environment of Mn impurities was basically uniform, which also made the doped FL show single exponential decay (Figure 8c,d). After further coating with a ZnS shell, the PLQYs of Mn:ZnSe/ZnS NCs reached 70 ± 5%.

Manganese-doped LHP NCs are one of the most studied and deeply investigated metal-doped LHP materials at present. Similar to traditional semiconductor NCs, the Mn impurity in LHP NCs can also produce the ^4^T_1_→^6^A_1_ characteristic PL. Liu et al. [71] believe that when the bandgap width (from 1.8 to 3.1 eV, from I^−^ to Cl^−^) and the energy difference Δ between the ^4^T_1_→^6^A_1_ transition (2.15 eV) of CsPbX_3_ NCs and the Mn impurity are greater than zero, effective forward energy transfer can occur between CsPbX_3_ NCs and the Mn impurity (Figure 8a). However, the host-dopant energy transfer (HDET) rate in LHP NCs is slower than that of II-VI semiconductor NCs [72], and both doped FL and band edge radiation can usually be observed simultaneously. The ratio of the FL intensities of the two is given by Equation (2) [73]:

(2) $$\frac{{\mathrm{PL}}_{\mathrm{Mn}}}{{\mathrm{PL}}_{\mathrm{eh}}}=\frac{k_{\mathrm{Mn},\mathrm{rad}}}{k_{\mathrm{ehn},\mathrm{rad}}}\frac{k_{\mathrm{ET}}}{k_{\mathrm{Mn}}+k_{\mathrm{Et}}e^{-\alpha ∆/kT}}$$

One of the key factors affecting the performance of LHP NCs is the presence of impurity atoms, such as Mn^2+^, which can act as electron donors or acceptors. The PL_Mn_ and PL_eh_ represent the FL intensities of doped FL and band-edge radiation, respectively, while k_Mn,rad_ and k_eh,rad_ represent the decay rate constants of doped FL and band-edge radiation, and k_ET_ is the rate of energy transfer from the band edge to the Mn impurity. Increasing the temperature will exacerbate non-radiative thermal relaxation and promote reverse energy transfer from the band edge to the Mn impurity, leading to an increase in the PL_Mn_/PL_eh_ ratio [74]. Recently, Wei et al. [75] have further proposed that the Mn impurity does not transfer energy directly from the band edge, but rather recovers non-radiative energy from defect levels and uses it for FL (Figure 8d). The photoluminescence quantum yield (PLQY) of doped FL can be tuned by changing the doping concentration, composition, and dimension, and the FL properties can also be controlled. Parobek et al. [76] synthesized CsPbCl_3_ NCs with a Mn^2+^ doping concentration of about 0.2%, with a PLQY range of 22–58% and a FL lifetime of 1–2 ms. By increasing the synthesis temperature or increasing the amount of Mn precursor, the doping concentration can be increased, thereby changing the intensity of doped FL and the FL lifetime. Liu et al. [77] prepared a series of CsPbxMn_1−x_Cl_3_ QDs with different doping concentrations up to 46%, and found that as the synthesis temperature increased, the relative FL intensity of doped FL to band-edge radiation first increased and then decreased, while the Mn emission peak wavelength shifted to longer wavelengths, attributed to the Mn–Mn interaction at high concentrations.

Compared with cadmium-based NCs, Mn^2+^-doped ZnS NCs (Mn:ZnS NCs) have lower toxicity and do not cause obvious damage to cells [78], so they are suitable for use as optical sensing probes after being rationally developed or structurally designed [79]. In the past five years, researchers have designed FL quenching-type probes based on principles such as electron transfer, inner filter effect, energy transfer, and surface ligand interactions, enabling effective detection of Tl^+^ [80], dopamine [81], clenbuterol (resorcinol) [82], and xanthine oxidase [83] using Mn:ZnS NCs. At the same time, FL enhancement-type probes have been designed using principles such as defect passivation and induced aggregation, enriching the detection of organophosphate pesticides [84], herbicide fomesafen [85], biochemical phosphate [86], and fish protein [87] using Mn:ZnS NCs. Today, FL sensing research based on Mn:ZnS NCs still has its unique vitality.

LHP NCs used as a novel optical oxygen sensor material is feasible. Recently, Chen et al. focused on a class of doped LHP NCs, namely, Mn_2_:CsPbX_3_ NCs formed by partially replacing the [PbX_6_]^4−^ octahedron in the perovskite structure with Mn^2+^, replacing Pb^2+^ [77,88,89,90]. When the conduction band bottom or electron defect state energy level of CsPbX_3_ (X = Cl^−^ or Br^−^) matches the lowest excited state energy level of Mn impurity ^4^T_1_, a process of exciton–Mn^2+^ exchange coupling occurs, leading to host–dopant energy transfer [73,75,76] and activating the forbidden ^4^T_1_→^6^A_1_ transition. Therefore, in addition to the exciton band edge FL of the perovskite host, the Mn^2+^ ions located in the octahedral coordination field will also emit FL at around 580 nm with a millisecond-level lifetime [91], laying the foundation for the FL oxygen sensing research of Mn^2+^:CsPbCl_3_ NCs. Lin et al. studied the oxygen sensing ability based on the energy transfer of luminescent dopants and host dopants, and observed that Mn^2+^-doped FL in Mn^2+^:CsPbCl_3_ can be used as a sensing signal for oxygen content [92]. There have also been studies showing that quantum cutting occurs in Mn^2+^:CsPbCl_3_, causing the Mn^2+^-related FL and surface defect state FL to occur simultaneously and be quenched by oxygen [93]. These studies demonstrate the good potential of LHPs as a new type of optical oxygen sensor material.

**Figure 9:**
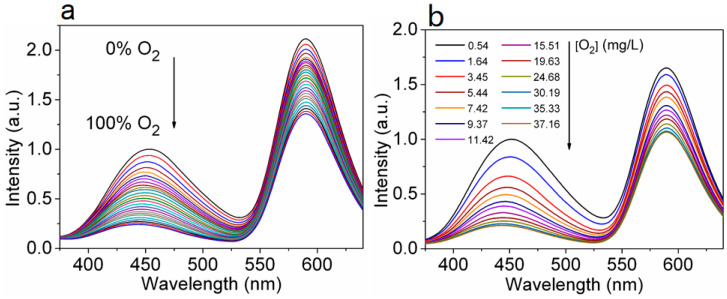
FL emission spectra of Mn:ZnS sensing film in (**a**) different O_2_ percentages and (**b**) different concentrations of dissolved oxygen. Excitation wavelength is 330 nm [94].

The research group [94] studied the effect of O_2_ on the FL of Mn(II)-doped CsPbCl_3_ (Mn:CsPbCl_3_ NCs) for the first time. By changing the quantity and distribution of Mn(II) to adjust the degree of energy transfer from perovskite to Mn(II) dopant, the authors concluded that O_2_ can temporarily interfere with the Mn(II) ligand field near the surface of Mn:CsPbCl_3_ NCs, thus affecting the emission process of Mn(II) ^4^T_1_→^6^A_1_. The results as shown in Figure 9 reveal that the FL intensity of the Mn:CsPbCl_3_ NC oxygen sensing film presents good linear correlations with the percentage of O_2_ gas in the range of 0–12% (Figure 9a), and the concentration of 0.54–11.42 mg/L for dissolved oxygen (Figure 9b). The responses are quickly reversible. These results suggest that oxygen sensing applications for light-emitting semiconductors are promising.

Doping is a process of purposefully introducing different elements into the main material and is an effective strategy for implanting novel optical properties in semiconductors. Since the 6*s* and 6*p* orbitals of Pb are involved in the formation of valence bands and conduction bands of LHP, substitutive doping of Pb^2+^ with isovalent or heterovalent metal ions will change the exciton recombination process of the main body of perovskite [71]. The types of metal ions that can be doped [95] include main group metal ions, transition metal ions, lanthanide metal ions and so on. At present, the most representative and widely studied doped FL in two-dimensional (2D) LHPs is Mn^2+^ FL, that is, ^4^T_1_→^6^A_1_ transition radiation. According to the crystal field theory, Mn^2+^ in the octahedral field has a low energy ^4^T excited state, which produces orange-red light emission. Mn^2+^ in the tetrahedral field has a ^4^T excited state with high energy, which produces green light emission [96]. In Mn^2+^-doped 2D LHP, the ^4^T_1_→^6^A_1_ transition produces emission wavelengths in the orange-red light region, and has a large Stokes shift and long lifetime (microsecond to millisecond). Because the splitting degree of the d electron level of Mn^2+^ is affected by the crystal field environment, there may be multiple emission levels at the same time, so the spectral broadening of Mn^2+^ emission usually occurs.

**Figure 10:**
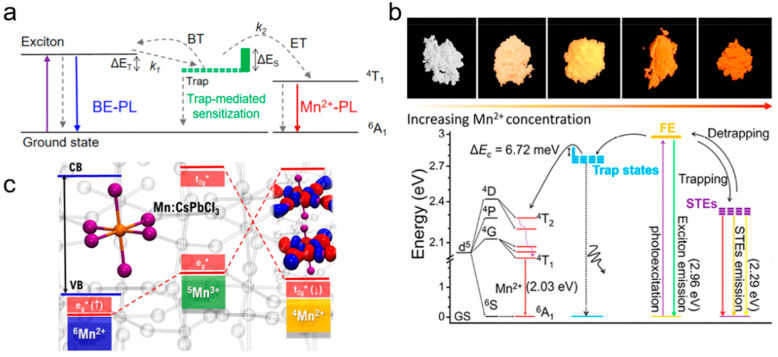
Mechanism diagrams of Mn^2+^ dopant emission based on (**a**,**b**) energy-transfer and (**c**) charge-transfer [101] pathways, where the asterisk represents the excited state.

The Mn^2+^ FL principle of 2D LHPs is basically the same as that of 3D counterparts (Figure 10). When Mn^2+^ replaces part of the Pb^2+^ in the octahedral field to form [MnCl_6_]^4−^, the d orbitals of Mn^2+^ interact with halogen orbitals with the same symmetry to produce bonding molecular orbitals and antibonding molecular orbitals. Since ^4^T_1_→^6^A_1_ is parity barred, the Mn^2+^ impurity produces a ^4^T_1_ excited state by transferring exciton energy from the perovskite body [73,77,88,97]. For 2D LHPs, the energy transfer rate is determined by the dynamics of photogenerated heat excitons [98]. Doping Mn^2+^ not only introduces new luminous energy levels, but also introduces a shallow trapping state to assist exciton energy transfer [75,99]. In addition to the widely reported energy transfer excitation pathway, existing mechanism studies also support an excitation pathway based on charge transfer [100,101]. The photogenerated holes of the main body of the LHP are captured by the ^6^Mn^2+^ ground state (^6^A_1_, t_2g_^3^e_g_^2^), removing electrons from the eg* (↑) state of the antibonding molecular orbital to form the oxidation intermediate state ^5^Mn^3+^, (t_2g_^3^e_g_^1^) and then accepting electrons in the t_2g_* (↓) state of the antibonding molecular orbital. The ^4^Mn^2+^ excited states (^4^T_1_, t_2g_^4^e_g_^1^) are produced by non-radiative decay. The charge transfer mechanism uses Mn itself as the trapping state, mediating ^4^T_1_ sensitization (^5^Mn^3+^), which needs to overcome a small energy barrier related to structural deformation, but avoids spin and orbital constraints, so it may be a more favorable excitation pathway.

Generally, the sensing mechanism of perovskite metal halides for oxygen is usually based on the quenching effect of oxygen molecules on the material’s FL. When oxygen molecules come into contact with the surface of perovskite, they capture the excited-state electrons, resulting in a decrease in FL intensity. By detecting the changes in FL intensity, the concentration of oxygen can be quantitatively analyzed. The following steps occur in the quenching-based oxygen sensing process: 1. FL quenching mechanism, where oxygen acts as a quencher and can interact with the excited state of perovskite metal halides, leading to a reduction in FL intensity; 2. dynamic quenching, where oxygen molecules diffuse to the surface of perovskite and undergo non-radiative energy transfer with the excited-state electron–hole pairs, reducing radiative recombination and thereby reducing FL intensity. In both of these forms, the concentration of oxygen and the degree of FL quenching show a linear relationship within a certain range.

For perovskite metal halides in FL-enhanced oxygen sensing, the sensitivity and response speed of oxygen sensing can be significantly improved by optimizing the structure of perovskite metal halides or performing surface modification. For example, introducing defect engineering, surface ligand modification, or doping with other elements can enhance the material’s adsorption capacity for oxygen, thereby improving the sensing performance for oxygen. Table 1 reveals the FL sensing situation for oxygen using perovskite metal halides.

**Table 1:** Oxygen sensing of perovskite metal halides.

Sensing Material	Sensing Mechanism	Comments	Reference
Nanocubes (NCs) of CsPbBr_3_ perovskite	FL quenching	Highlights the limits of the defect tolerance argument in 1D, 2D and bulk perovskite systems, while confirming the good optical performances exhibited by the better passivated NCs; reveals the pivotal role of the surface structure in the optical properties of inorganic perovskite systems.	[60]
Nanowires (NWs) of CsPbBr_3_ perovskite	FL enhancement		
Nanosheets (NSs) of CsPbBr_3_ perovskite	FL enhancement		
Bulk single crystals (SCs) of CsPbBr_3_ perovskite	FL enhancement		
MAPbI_3_	FL enhancement	Reversible; the oxidation of interstitial iodine is favored over that of lattice iodine, effectively inactivating a source of deep traps with levels located in the MAPbI_3_ band gap.	[62]
MAPbX_3_ NCs	FL quenching	The role of oxygen during the photodegradation process as an oxygen-assisted lightinduced etching, which results in a blue shift in the FL peak position due to NC size reduction and eventually complete FL vanishing.	[63]
Mn:CsPbCl_3_ NCs	FL quenching	Sensing range from 0 to 100%; good linear response in the 0−12% O_2_ concentration range; high sensing reversibility and rapid signal response.	[92]
Mn^2+^-doped CsPbCl_3_ PQDs	FL quenching	An undocumented, oxygen-sensitive, and broad emission band (915−1150 nm) emerges peaking at around 950 nm; the emission band at 950 nm possibly originates from Mn^2+^-associated defect states.	[93]

## 5. FL Properties of Copper-Doped NCs

**Figure 11:**
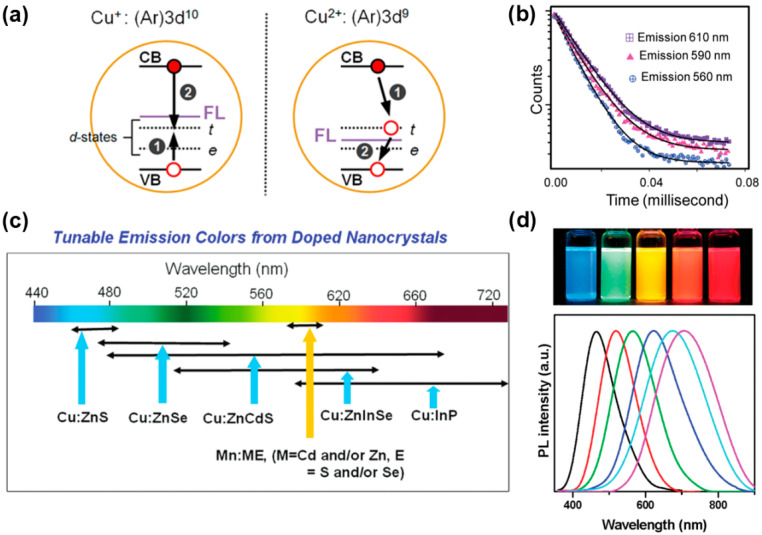
(**a**) Schematic illustration of exciton recombination process of Cu-doped NCs [102]; (**b**) dopant emission decay curves at different emission centers [103]; (**c**) the range of FL emission of some Cu-doped semiconductor NCs [104]; (**d**) FL emission spectra of Cu:ZnS/Zn_1−x_Cd_x_S NCs under different Cd/Zn precursor ratios, and the digital picture of samples under UV light illumination [108].

Another case of semiconductor nanocrystalline doping FL is the electron–hole recombination process involving both the impurity and the host nanocrystalline after the photoexciton is transferred to the impurity level, such as Cu doping. Brovelli et al. [102] found through experimental study and calculation that there are two possible valence states of Cu impurity in d-dots, +1 and +2, and the impurity level related to Cu^+^ (3d^10^) can radiatively recombine with conduction band electrons after trapping holes from the valence band. The impurity levels associated with Cu^2+^ (3d^9^) have properties similar to permanent holes, so the conduction band electrons are captured first for radiative recombination, and then the electrons return to the valence band. Both cases produce Cu-doped FL (Figure 11a). Similar to Mn-doped FL, the spectral broadening of the Cu emission peak is also large. Srivastava et al. [103] believe that valence band hole transfer causes the existence of two d electron levels T_2_ and E at the same time after Cu^+^ is transformed into Cu^2+^, and multi-level FL causes the spectral broadening. In addition, the fluorescence lifetime of Cu-doped FL usually ranges from a few hundred nanoseconds to a few microseconds, depending on the variation in the host NCs (Figure 11b).

Changing the composition and size of d-dots can achieve a continuous shift in the Cu-doped FL wavelength from the visible to the near-infrared region (Figure 11c) [104]. With the increase in host NCs size, the doped FL wavelength of Cu:ZnSe NCs is in the range of 470–550 nm [105], and the emission spectrum wavelength of Cu:InP NCs can be significantly redshifted from 630 nm to 1100 nm [106]. Srivastava et al. [103] prepared alloyed Cu:ZnS/Zn_1−x_Cd_x_S NCs with a sphalerite structure, and the wavelength of Cu-doped FL can be redshifted from 500 nm to 680 nm. Sarkar et al. [107] prepared alloyed Cu:Zn-In-Se NCs with an ultra-small size (less than 2.5 nm) and good photostability, and changed the composition ratio of In^3+^ and Zn^2+^ through surface cation exchange, which not only maintained the original particle size of the host NCs but also changed the band gap width. Thus, the wavelength of Cu-doped FL is blue shifted in the range of 660–540 nm. Zhang et al. [108] synthesized Cu:ZnS/Zn_1−x_Cd_x_S NCs using the one-pot method. By adjusting the ratio of Cd and Zn precursors, doped FL covering 440–710 nm was obtained without changing the nanocrystalline size (Figure 11d), and it also has good thermal stability at 250 °C.

Currently, Cu-doped perovskite NCs with Cu as the B-site ion are more common in non-lead 2D perovskite materials and double perovskites, possibly because Cu^2+^ has a smaller ionic radius than Pb^2+^, which is not conducive to the formation of structurally stable ABX_3_ LHP NCs. In addition, Cu^2+^ can introduce defects into LHP NCs [109] or transfer its photogenerated electrons [110], so it is not conducive to FL.

**Figure 12:**
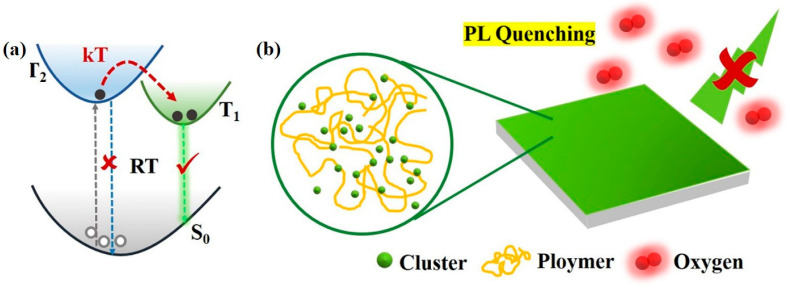
(**a**) FL pathway diagram of Cu_4_I_4_(PPh_3_)_4_; (**b**) sensing mechanism [113].

In semiconductor luminescent materials, copper–iodine clusters have a relatively long luminescent lifetime, which makes their three-wire excited states very easy to exchange energy with external materials. Similar to the excited states of copper–iodine clusters, oxygen molecules also exhibit trilinear states under normal conditions, so the sensing of copper–iodine clusters on oxygen can be studied based on this property. In recent years, researchers have found that some copper-based organic complexes with molecular formula [Cu(N-N)(P-P)] or [Cu(N-N)(L)_2_] (N-N, P-P and L represent nitrogen-containing bidentate ligands, phosphine-containing bidentate ligands and phosphine-containing monodentate ligands, respectively) have oxygen sensing capabilities [111,112,113]. Zhang et al. [113] prepared a sensing film by mixing Cu_4_I_4_(PPh_3_)_4_ with polystyrene using the intrinsic FL properties of Cu_4_I_4_ copper–iodine clusters. They studied the sensing mechanism and the FL pathway is shown in Figure 12. The test results show that the sensor film has a good response to the change in oxygen concentration, and its maximum FL quenching ratio is 5.4. This study opens the application of Cu_4_I_4_ copper–iodine clusters in the field of oxygen sensing. The effects of different substituents on the oxygen sensing behavior of Cu(I) complexes were further studied, and it was found that the electron-withdrawing substituents were conducive to increasing the FL lifetime of the complexes, thereby improving the oxygen sensitivity [114]. The machinability of the organic part makes it possible to adjust the sensing performance of the metal–organic complex oxygen-sensitive probe, demonstrating the advantages of the metal–organic complex probe.

The four-core copper iodide hybrid materials are a kind of classic organic and inorganic hybrid materials that are favored in crystal engineering. On the one hand, the diversity of its structure comes from the diversity of inorganic units formed by cuprous iodide, and on the other hand, it comes from different organic ligands that have a guiding effect on the formation of inorganic units. The low energy emission bands of these materials at room temperature are closely related to the Cu–Cu interaction, indicating that they are independent of organic ligands. Recently, Huitorel Brendan et al. [115] evaluated the influence of different phosphine-containing ligands on the photophysical properties of tetralane-type copper iodide hybrid materials, and synthesized different tetralene copper iodide hybrid materials by modifying substituent groups (-OCH_3_, -CH_3_, -CF_3_) with the para-position of the benzene ring in the triphenylphosphine ligand. The results show that the hybrid materials with electron-donating substituents still exhibit classical cluster core centered FL, emitting yellow and yellow-green light at room temperature, while the hybrid materials with strong electron-absorbing substituents exhibit ligand-related FL properties, showing blue and white light at room temperature. By theoretical calculation, the existence of electron-withdrawing substituents leads to the reduction in the lowest molecular unoccupied orbital (LUMO) energy, which affects the luminous properties of hybrid materials. In addition, the researchers say that the importance of the form of the material’s crystal accumulation on the influence of photophysical properties cannot be ignored. Similarly, Xu et al. [116] designed a ligand-activated copper iodide hybrid cluster Cu_4_I_4_L_4_ to produce the first blue organic light-emitting diode device based on the cluster material. The introduction of electron-donating groups can effectively enhance the emission of ligand-related excited states, and eliminate the excited states centered on cluster nuclei by delocalizing electrons from cluster nuclei to organic ligands. All the above studies demonstrate the feasibility of regulating the photophysical properties of the four-core copper iodide hybrid materials by changing the ligands.

**Figure 13:**
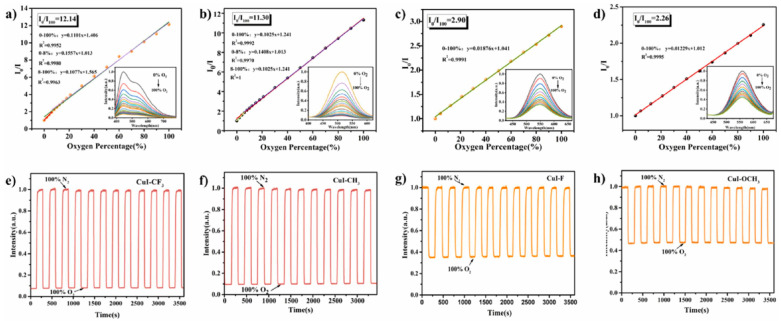
Stern–Volmer plots of different tetranuclear copper iodide hybrid materials towards oxygen sensing (inserted graph: the corresponding emission spectra): (**a**) CuI-CF_3_; (**b**) CuI-CH_3_; (**c**) CuI-F; (**d**) CuI-OCH_3_, the Stern–Volmer plot of CuI-CF_3_ was obtained by calculating the emission intensity at 443 nm. Cyclic curve of different tetranuclear copper iodide hybrid materials under alternating N_2_ and O_2_ atmosphere within one hour: (**e**) CuI-CF_3_; (**f**) CuI-CH_3_; (**g**) CuI-F; (**h**) CuI-OCH_3_ [117].

The sensitivity of type I copper–iodine clusters to oxygen was improved by ligand modification [117]. A CuI-CF_3_ copper–iodine cluster CuI-CF_3_ was prepared using the one-pot method at room temperature by mixing triphenylphosphine ligands modified by -CF_3_ with Cu porous iodide. The Cui-CF_3_/PS sensor film was prepared by mixing CuI-CF_3_ with polystyrene (PS), and its oxygen response performance was investigated. The FL intensity of the materials with -CF_3_ ligands can be fully quenched by gaseous oxygen, and the sensitivity expressed by the maximum FL quench ratio I_0_/I is 10.6. The hydrophobicity of PS endows the sensor film with excellent water resistance, which means that it can be used for the detection of dissolved oxygen content in water. The results show that the sensor film shows excellent performance in both gaseous and dissolved oxygen detection, that is, full range linearity (0–100%), fast response, and multiple cycles. The good performance of CuI-CF_3_ in gaseous oxygen and dissolved oxygen sensing tests confirms that the ligand modification strategy can effectively improve the sensing sensitivity of Cu_4_I_4_ copper–iodine clusters, which has great potential to become a highly sensitive oxygen sensor (Figure 13). The FL properties of type II copper–iodine clusters were enhanced by introducing a Mn(II) FL center and the oxygen sensing behavior of the clusters was adjusted by changing the crystal field environment of Mn(II). All the prepared sensing films showed a linear response in the range of 0–90% gaseous oxygen content, and maintained good FL reversibility after 1 h of nitrogen and oxygen cycle purging. Mn(II) coordinated by dppeO_2_ has a significantly higher oxygen response than dppeO_2_. The electron paramagnetic resonance (EPR) and time-resolved spectroscopy (TMS) tests show that the proportion of electrons in the ^4^T_1_ excited state is different due to the different crystal field intensity, which results in the different degree of oxygen quenching of the clusters. In addition, the nature of the ionic bond provides good stability to the material, making it also successful for the detection of dissolved oxygen content.

In addition, the self-assembly of organic ligands and zero-dimensional copper iodide inorganic cluster nuclei to form extended networks is a very good strategy for constructing functional materials [118], which can combine the photophysical properties of copper iodide materials with the advantages of the assembly structure at the molecular level. Metal–organic frameworks (MOFs) have the advantages of high porosity, low density, adjustable pore size and diversity of topological structures. The use of Cu_2_I_2_ and Cu_4_I_4_ cluster nuclei as secondary building units to design crystalline porous materials with ideal topological structures has potential applications in optical sensing and even colorimetric sensing. There are still relatively few reports on CuI-MOF synthesis. Although copper iodide hybrid materials have excellent properties, research in this field has been limited to the molecular structure level and its corresponding luminous properties, ignoring the impact of size effect on material properties. In the past two years, researchers began to devote themselves to the synthesis of nanoscale copper iodide hybrid materials and discovered new luminous properties, opening up another research direction. The rich structural chemistry, excellent optical properties and machinability of CuI-based hybrid materials provide a platform for the development of new photoelectric materials and composites. Although research in this area is far from sufficient, given the high abundance, low cost and low toxicity of copper salts, the development of new photoelectric materials from these substances is a very attractive option from an industrial and technical point of view.

Copper iodide hybrid materials give FL emission when excited by light of specific wavelengths, and their FL intensity is closely related to the structure of the materials, ligands and the environment. Due to the quenching effect of oxygen, oxygen can transfer energy or electrons with the excited copper iodide clusters, resulting in non-radiative energy transfer and thus weakening or completely quenching the FL intensity. This dynamic quenching effect is directly related to the oxygen concentration. When oxygen comes into contact with copper iodide clusters, the FL intensity decreases with the increase in oxygen concentration. By detecting the change in FL intensity, the oxygen concentration can be calculated. Table 2 reveals the FL sensing situation for oxygen using copper iodide hybrid materials.

## 6. Conclusions and Perspectives

A large number of optical oxygen sensors based on organic fluorophores or transition metal complexes have been developed in recent years. Due to the lack of light stability of the former, although oxygen sensors are mainly based on metal complexes or metal porphyrins as sensing materials, the expensive price and potential heavy metal toxicity of such materials still need to be further improved and improved.

The development of semiconductor luminescent materials and their application in oxygen sensing have provided new methods and approaches to solve the above problems. Among these semiconductor luminescent sensing materials, perovskite metal halides and copper iodide cluster luminescent materials have shown good sensitivity and response rate to oxygen sensing, with potential application value. This paper provides a systematic review of the recent developments of these two types of semiconductor materials for oxygen sensing. At present, the research on optical sensing of perovskite metal halides mainly follows three main directions: new material development, exploration of sensing mechanisms, and high-performance response applications. By designing the composition and structure of these materials from the source, simplifying their synthesis methods, optimizing their sensing performance, and analyzing sensing mechanisms, a new class of easy-to-synthesize and cost-effective optical oxygen sensing materials has been developed, with the goal of achieving high sensitivity, wide detection range, rapid and reversible response to oxygen. From the perspectives of material design, synthesis, and optical properties, further research on transition metal-doped two-dimensional lead halide perovskites in oxygen sensing can be conducted, developing an oxygen sensing method based on the ^4^T_1_→^6^A_1_ FL. In addition, the research can be conducted from the following aspects: (1) metal halide perovskites are inherently unstable in polar solvents, including water, even with surface organic ligands, which is especially prominent in 3D perovskite nanomaterials, limiting the determination of dissolved oxygen content. This problem is currently being solved through various encapsulation strategies, such as surface modification with block copolymers or phospholipids, encapsulation with micelles using hydrophobic polymers or silica-based materials, and encapsulation of individual particles or the entire particle using hydrophobic polymers or silica-based materials. However, the new problem is that encapsulation reduces the degree of contact between the perovskite and the target material, which may partially reduce the sensing speed and sensitivity. Therefore, the balance between the stability of the perovskite and the sensing performance needs to be continuously adjusted by adjusting the materials and methods of encapsulation. In the future, low-dimensional metal halide perovskites with more hydrophobic components can be developed to improve the stability of the material from the source, while not affecting the direct contact between the target material and the perovskite. Screening more hydrophobic organic components or conducting surface coating treatment that is both breathable and waterproof can expand the water-phase oxygen sensing application. (2) The interaction mechanism between oxygen molecules and perovskite sensing materials can be analyzed from the perspective of optical properties, which can explain the oxygen sensitivity of doped FL from more crystal structures and theoretical calculations, and uncover more potential oxygen sensing materials. (3) The sensing performance of metal halide perovskites is limited by both their composition structure and the fabrication process. By combining more advanced materials with superior performance, such as metal–organic frameworks or molecularly imprinted polymers, and developing composite sensing materials based on metal halide perovskites, the sensing performance can be enriched from a chemical perspective. From the perspective of process stages, the current perovskite sensing research is mostly at the laboratory stage. In order to improve the efficiency of sensing material preparation and reduce the influence of operation factors on sensing performance, more convenient and batch synthesis methods for high-quality perovskites need to be proposed in the future. At the same time, efforts should be made to promote the deviceization of sensing systems, thus establishing more practical sensing platforms based on perovskite luminescent materials. To enhance the practicality of perovskite oxygen sensing materials, the sensing system can be deviceized, including printing optical oxygen sensing arrays or oxygen sensing fibers, and combined with the above first measure to build miniaturized sensors that can be applied in more environmental conditions.

Copper iodide hybrid materials are a class of low-cost, easy-to-synthesize, and superior light-emitting materials. The unique structure of copper iodide hybrid materials endows them with a variety of stimulus-responsive behaviors, while the presence of different nuclear numbers of inorganic cores and organic ligands allows for the fine-tuning of the material’s luminescent properties, enabling the design of various dimensional copper-based hybrid materials. Therefore, these materials are a class of functional luminescent materials with great development prospects and practical value. Researchers have focused on the crystal structure of copper iodide hybrid materials, but there has been little progress in their practical applications, mainly due to the rigid framework formed by the inorganic cores of cuprous iodide and the poor dispersibility and solubility of the material. The predominant form of the material in the form of a block structure limits its further application. Most of the literature reports are situated within the field of luminescent phosphor and organic light-emitting diodes, while there are few reports in the field of analytical sensing and biological imaging. Therefore, expanding the application of this material in the field of analytical sensing deserves attention. Zero-dimensional tetranuclear copper iodide hybrid materials, as the most widely studied branch of copper iodide hybrid materials, have higher structural stability and optical stability than other structures, and have better potential for application in the field of analytical sensing than other structures. Their triplet FL property also makes tetranuclear copper iodide hybrid materials potentially applicable in the field of analytical sensing.

Copper iodide clusters are also gaining attention in the field of optical semiconductors due to their low toxicity and abundant sources. In this family of materials, type I copper iodide clusters, which are composed of coordination bonds, generally have strong FL properties. Among them, the inorganic module with the structure of Cu_4_I_4_ demonstrates stimulus-responsive behavior, making it suitable for research in the field of analytical sensing. Type II copper iodide clusters, which are composed of ionic bonds, have excellent structural stability and solvent processability, but their application in photochemical oxygen sensing is limited by their insufficient FL performance, and further strategies need to be developed to improve their FL, opening up the application of these materials in photochemical oxygen sensing.

In addition to the aforementioned perovskite metal halides, transition metal-doped two-dimensional lead halide perovskites, copper iodide clusters and copper iodide hybrid materials, some novel luminescent materials have emerged recently, such as carbon quantum dots (CQDs). The FL quenching of CQDs by oxygen [4] or reactive oxygen species produced by biological reactions [119,120] is utilized for oxygen FL sensing. The research and application of these materials in oxygen sensing detection may also lead to new directions and approaches.

## Figures and Tables

**Table 2:** Oxygen sensing of copper iodide hybrid materials.

Sensing Material	Sensing Mechanism	Sensing Range	Sensitivity *I*_0_/*I*_100_	Response Time	Reference
[Cu(POP)(PTZ)]BF_4_	FL quenching	0–100%	11.16	5 s	[112]
Cu_4_I_4_(PPh_3_)_4_/PS film	FL quenching	0–100% (gas)	5.4	8 s	[113]
		0.9–34.06 mg/L (dissolved oxygen)	4.8	145 s	
[Cu(L)(PPh_3_)_2_]BF_4_/PS fibrous films	FL quenching	0–100%	15.81	6 s	[114]
CuI-CF_3_	FL quenching	0–100%	12.14	-	[117]
[Mn(dppmO_2_)_3_]Cu_2_I_4_·3MeCN	FL quenching	0–90%	6.6	9 s	[118]
[Mn(dppeO_2_)_3_]Cu_2_I_4_	FL quenching	0–90%	2.1	13 s	

## References

[1] Wang X.D., Wolfbeis O.S. (2014). Optical Methods for Sensing and Imaging Oxygen: Materials, Spectroscopies and Applications. Chem. Soc. Rev..

[2] Xu Y.H., Qi G.P., Hu D.S. (2022). Development of Dissolved Oxygen Sensor Based on Time-Domain Lifetime Measurement with a Sensing Film Fabricated by Embedding PtOEP in Highly Stable and Highly Hydrophobic Fluorinated Matrix. Chem. Asian J..

[3] Bian Z., Chao C., Feng S. (2021). Dissolved Oxygen Sensing Characteristics of Plastic Optical Fiber Coated with Hydrogel Film. Opt. Fiber Technol..

[4] Zhang Y., Yang H., Gao W. (2024). Research Progress of Optical Dissolved Oxygen Sensors: A Review. IEEE Sens. J..

[5] Xiong X., Xiao D., Choi M.M.F. (2006). Dissolved Oxygen Sensor Based on Fluorescence Quenching of Oxygen-Sensitive Ruthenium Complex Immobilized on Silica–Ni–P Composite Coating. Sens. Actuators B-Chem..

[6] Kneas K.A., Xu W., Demas J.N. (1998). Luminescence-Based Oxygen Sensors: ReL(CO)_3_Cl and ReL(CO)_3_CN Complexes on Copolymer Supports. J. Fluoresc..

[7] Mak C.S.K., Pentlehner D., Stich M. (2009). Exceptional Oxygen Sensing Capabilities and Triplet State Properties of Ir(ppy-NPh_2_)_3_. Chem. Mater..

[8] Qi X.L., Liu S.Y., Lin R.B. (2013). Phosphorescence Doping in a Flexible Ultramicroporous Framework for High and Tunable Oxygen Sensing Efficiency. Chem. Commun..

[9] Xu W., Kneas K.A., Demas J.N. (1996). Oxygen Sensors Based on Luminescence Quenching of Metal Complexes: Osmium Complexes Suitable for Laser Diode Excitation. Anal. Chem..

[10] Lan G., Ni K., You E. (2019). Multifunctional Nanoscale Metal–Organic Layers for Ratiometric pH and Oxygen Sensing. J. Am. Chem. Soc..

[11] Xu R., Wang Y., Duan X. (2016). Nanoscale Metal–Organic Frameworks for Ratiometric Oxygen Sensing in Live Cells. J. Am. Chem. Soc..

[12] Chu C.S., Lo Y.L. (2007). High-Performance Fiber-Optic Oxygen Sensors Based on Fluorinated Xerogels Doped with Pt(II) Complexes. Sens. Actuators B-Chem..

[13] Huo C., Zhang H., Zhang H. (2006). Synthesis and Assembly with Mesoporous Silica MCM-48 of Platinum(II) Porphyrin Complexes Bearing Carbazyl Groups:  Spectroscopic and Oxygen Sensing Properties. Inorg. Chem..

[14] Zhang K.Y., Gao P., Sun G., Zhang T., Li X., Liu S., Zhao Q., Lo K.K.-W., Huang W. (2018). Dual-Phosphorescent Iridium(III) Complexes Extending Oxygen Sensing from Hypoxia to Hyperoxia. J. Am. Chem. Soc..

[15] Wolfbeis O.S. (2015). Luminescent Sensing and Imaging of Oxygen: Fierce Competition to the Clark Electrode. Bioessays.

[16] Lorenzon M., Pinchetti V., Bruni F. (2017). Single-Particle Ratiometric Pressure Sensing Based on “Double-Sensor” Colloidal Nanocrystals. Nano Lett..

[17] Hu Z., Liu S., Qin H. (2020). Oxygen Stabilizes Photoluminescence of CdSe/CdS Core/Shell Quantum Dots Via Deionization. J. Am. Chem. Soc..

[18] Basu B.J., Anandan C., Rajam K.S. (2003). Study of the Mechanism of Degradation of Pyrene-Based Pressure Sensitive Paints. Sens. Actuators B-Chem..

[19] Basu B.J., Thirumurugan A., Dinesh A.R., Anandan C., Rajam K.S. (2005). Optical Oxygen Sensor Coating Based on the Fluorescence Quenching of a New Pyrene Derivative. Sens. Actuators B-Chem..

[20] Yan X., Peng H., Xiang Y., Wang J., Yu L., Tao Y., Li H.H., Huang W., Chen R.F. (2022). Recent Advances on Host-Guest Material Systems toward Organic Room Temperature Phosphorescence. Small.

[21] Feng G.X., Zhang G.Q., Ding D. (2020). Design of Superior Phototheranostic Agents Guided by Jablonski Diagrams. Chem. Soc. Rev..

[22] Keruckiene R., Kusas N., Dvylys L., Skuodis E., Matulis V.E., Ragoyja E.G., Lyakhov D.A., Klymenko I., Grazulevicius J.V. (2022). Derivatives of Bis(Trifluoromethyl)Biphenyl and Various Donor Noieties Exhibiting Dual State Emission. J. Lumin..

[23] Keruckiene R., Volyniuk D., Leitonas K., Grazulevicius J.V. (2020). Dual Emission Fluorescence/Room-Temperature Phosphorescence of Phenothiazine and Benzotrifluoride Derivatives and Its Application for Optical Sensing of Oxygen. Sens. Actuators B-Chem..

[24] Liao Q.Y., Gao Q.H., Wang J.Q., Gong Y.B., Peng Q., Tian Y., Fan Y.Y., Guo H.J., Ding D., Li Q.Q. (2020). 9,9-Dimethylxanthene Derivatives with Room-Temperature Phosphorescence: Substituent Effects and Emissive Properties. Angew. Chem. Int. Ed..

[25] Cai S.Z., Shi H., Tian D., Ma H.L., Cheng Z., Wu Q., Gu M.X., Huang L., An Z.F., Peng Q. (2018). Enhancing Ultralong Organic Phosphorescence by Effective π-Type Halogen Bonding. Adv. Funct. Mater..

[26] Skhirtladze L., Leitonas K., Bucinskas A., Woon K.L., Volyniuk D., Keruckiene R., Mahmoudi M., Lapkowski M., Ariffin A., Grazulevicius J. (2023). Turn on of Room Temperature Phosphorescence of Donor-Acceptor-Donor Type Compounds via Transformation of Excited States by Rigid Hosts for Oxygen Sensing. Sens. Actuators B-Chem..

[27] Bolton O., Lee K., Kim H.J., Lin K.Y., Kim J. (2011). Activating Efficient Phosphorescence from Purely Organic Materials by Crystal Design. Nat. Chem..

[28] Zhong W., Urayama P., Mycek M.A. (2003). Imaging Fluorescence Lifetime Modulation of a Ruthenium-Based Dye in Living Cells: The Potential for Oxygen Sensing. J. Phys. D Appl. Phys..

[29] Dobrucki J.W. (2001). Interaction of Oxygen-Sensitive Luminescent Probes Ru(Phen)_3_ and Ru(Bipy)_3_ with Animal and Plant Cells in Vitro: Mechanism of Phototoxicity and Conditions for Non-Invasive Oxygen Measurements. J. Photochem. Photobiol. B.

[30] DiMarco G., Lanza M., Pieruccini M., Campagna S. (1996). A Luminescent Iridium(III) Cyclometallated Complex Immobilized in a Polymeric Matrix as a Solid-State Oxygen Sensor. Adv. Mater..

[31] Zhou C., Zhao W.X., You F.T., Geng Z.X., Peng H.S. (2019). Highly Stable and Luminescent Oxygen Nanosensor Based on Ruthenium-Containing Metallopolymer for Real-Time Imaging of Intracellular Oxygenation. ACS Sens..

[32] Luo J.D., Xie Z.L., Lam J.W.Y., Cheng L., Chen H.Y., Qiu C.F., Kwok H.S., Zhan X.W., Liu Y.Q., Zhu D.B. (2001). Aggregation-Induced Emission of 1-Methyl-1,2,3,4,5-Pentaphenylsilole. Chem. Commun..

[33] Xing Y., Wang L., Liu C., Jin X. (2020). Effects of Fluorine and Phenyl Substituents on Oxygen Sensitivity and Photostability of Cyclometalated Platinum(II) Complexes. Sens. Actuators B-Chem..

[34] Ma H.L., Shen K., Wu Y.P., Xia F., Yu F.L., Sun Z.Y., Qian C.Y., Peng Q.M., Zhang H.H., You C. (2019). High-Color-Purity and Efficient Solution-Processable Blue Phosphorescent Light-Emitting Diodes with Pt(II) Complexes Featuring 3ππ* Transitions. Mater. Chem. Front..

[35] Liu C., Song X.L., Rao X.F., Xing Y., Wang Z.G., Zhao J.Z., Qiu J.S. (2014). Novel Triphenylamine-Based Cyclometalated Platinum(II) Complexes for Efficient Luminescent Oxygen Sensing. Dye. Pigment..

[36] Di L., Xia Z.Q., Wang H.H., Xing Y., Yang Z.X. (2021). Switchable and Adjustable Aie Activity of Pt(II) Complexes Achieving Swift-Responding and Highly Sensitive Oxygen Sensing. Sens. Actuators B-Chem..

[37] Qi J., Li J., Liu R.H., Li Q., Zhang H.K., Lam J.W.Y., Kwok R.T.K., Liu D.B., Ding D., Tang B.Z. (2019). Boosting Fluorescence-Photoacoustic-Raman Properties in One Fluorophore for Precise Cancer Surgery. Chem.

[38] Zhao K.Y., Mao H.T., Wen L.L., Shan G.G., Fu Q., Sun H.Z., Su Z.M. (2018). A Simple Strategy to Achieve Remarkable Mechanochromism of Cationic Ir(III) Phosphors through Subtle Ligand Modification. J. Mater. Chem. C.

[39] Che W.L., Li G.F., Liu X.M., Shao K.Z., Zhu D.X., Su Z.M., Bryce M.R. (2018). Selective Sensing of 2,4,6-Trinitrophenol (TNP) in Aqueous Media with “Aggregation-Induced Emission Enhancement” (AIEE)-Active Iridium(III) Complexes. Chem. Commun..

[40] Amao Y., Okura I. (2009). Optical Oxygen Sensor Devices Using Metalloporphyrins. J. Porphyr. Phthalocyanines.

[41] Wang X.D., Chen X., Xie Z.X. (2008). Reversible Optical Sensor Strip for Oxygen. Angew. Chem. Int. Ed..

[42] Zach P.W., Freunberger S.A., Klimant I., Borisov S.M. (2017). Electron-Deficient near-Infrared Pt(II) and Pd(II) Benzoporphyrins with Dual Phosphorescence and Unusually Efficient Thermally Activated Delayed Fluorescence: First Demonstration of Simultaneous Oxygen and Temperature Sensing with a Single Emitter. ACS Appl. Mater. Interfaces.

[43] Penso C., Rocha J.L., Martins M. (2021). PtOEP–PDMS-Based Optical Oxygen Sensor. Sensors.

[44] Zhang H., Zhang Z. (2020). Ratiometric Sensor Based on PtOEP-C6/Poly (St-TFEMA) Film for Automatic Dissolved Oxygen Content Detection. Sensors.

[45] Wu W.X., Su J., Tang C.C. (2017). CybLuc: An Effective Aminoluciferin Derivative for Deep Bioluminescence Imaging. Anal. Chem..

[46] Xia T.T., Cheng X.T., Zhan W.J. (2021). Activity-Based Luciferase-Luciferin Bioluminescence System for Bioimaging Applications. Anal. Sens..

[47] Yuan Z.H., Jiang Q.C., Liang G.L. (2025). Inspired by Nature: Bioluminescent Systems for Bioimaging Applications. Talanta.

[48] Jang Y., Shapiro A., Isarov M. (2017). Interface Control of Electronic and Optical Properties in IV-VI and II-VI Core/Shell Colloidal Quantum Dots: A Review. Chem. Commun..

[49] Bruchez M., Moronne M., Gin P., Weiss S., Alivisatos A.P. (1998). Semiconductor Nanocrystals as Fluorescent Biological Labels. Science.

[50] Chan W.C.W., Nie S. (1998). Quantum Dot Bioconjugates for Ultrasensitive Nonisotopic Detection. Science.

[51] Freeman R., Willner I. (2012). Optical Molecular Sensing with Semiconductor Quantum Dots (QDs). Chem. Soc. Rev..

[52] Wu P., Zhao T., Wang S. (2014). Semicondutor Quantum Dots-Based Metal Ion Probes. Nanoscale.

[53] Zhou J., Yang Y., Zhang C. (2015). Toward Biocompatible Semiconductor Quantum Dots: From Biosynthesis and Bioconjugation to Biomedical Application. Chem. Rev..

[54] Koren K., Hutter L., Enko B. (2013). Tuning the Dynamic Range and Sensitivity of Optical Oxygen-Sensors by Employing Differently Substituted Polystyrene-Derivatives. Sens. Actuators B-Chem..

[55] Li H.Y., Zhao S.N., Zang S.Q. (2020). Functional Metal–Organic Frameworks as Effective Sensors of Gases and Volatile Compounds. Chem. Soc. Rev..

[56] McGee K.A., Veltkamp D.J., Marquardt B.J. (2007). Porous Crystalline Ruthenium Complexes Are Oxygen Sensors. J. Am. Chem. Soc..

[57] McGee K.A., Mann K.R. (2009). Inefficient Crystal Packing in Chiral [Ru(phen)_3_](PF_6_)_2_ Enables Oxygen Molecule Quenching of the Solid-State MLCT Emission. J. Am. Chem. Soc..

[58] Wang X., Veerappan V., Cheng C. (2010). Free Solution Hydrodynamic Separation of DNA Fragments from 75 to 106,000 Base Pairs in A Single Run. J. Am. Chem. Soc..

[59] Lorenzon M., Sortino L., Akkerman Q. (2017). Role of Nonradiative Defects and Environmental Oxygen on Exciton Recombination Processes in CsPbBr_3_ Perovskite Nanocrystals. Nano Lett..

[60] Roda C., Abdelhady A.L., Shamsi J. (2019). O_2_ as a Molecular Probe for Nonradiative Surface Defects in CsPbBr_3_ Perovskite Nanostructures and Single Crystals. Nanoscale.

[61] Stoeckel M.A., Gobbi M., Bonacchi S. (2017). Reversible, Fast, and Wide-Range Oxygen Sensor Based on Nanostructured Organometal Halide Perovskite. Adv. Mater..

[62] Meggiolaro D., Mosconi E., De Angelis F. (2017). Mechanism of Reversible Trap Passivation by Molecular Oxygen in Lead-Halide Perovskites. ACS Energy Lett..

[63] Liu L., Deng L., Huang S. (2019). Photodegradation of Organometal Hybrid Perovskite Nanocrystals: Clarifying the Role of Oxygen by Single-Dot Photoluminescence. J. Phys. Chem. Lett..

[64] Xia Y.S., Zhang T.L., Diao X.L., Zhu C.Q. (2007). Measurable Emission Color Change: Size-Dependent Reversible Fluorescence Quenching of Cdte Quantum Dots by Molecular Oxygen. Chem. Lett..

[65] Li J., Wang H., Li D. (2020). Self-Trapped Excitons in Two-Dimensional Perovskites. Front. Optoelectron..

[66] Hu T., Smith M.D., Dohner E.R. (2016). Mechanism for Broadband White-Light Emission from Two-Dimensional (110) Hybrid Perovskites. J. Phys. Chem. Lett..

[67] Dohner E.R., Hoke E.T., Karunadasa H.I. (2014). Self-Assembly of Broadband White-Light Emitters. J. Am. Chem. Soc..

[68] Dohner E.R., Jaffe A., Bradshaw L.R. (2014). Intrinsic White-Light Emission from Layered Hybrid Perovskites. J. Am. Chem. Soc..

[69] Hazarika A., Pandey A., Sarma D.D. (2014). Rainbow Emission from an Atomic Transition in Doped Quantum Dots. J. Phys. Chem. Lett..

[70] Pu C., Ma J., Qin H. (2016). Doped Semiconductor-Nanocrystal Emitters with Optimal Photoluminescence Decay Dynamics in Microsecond to Millisecond Range: Synthesis and Applications. ACS Cent. Sci..

[71] Liu G., Qiu C., Tian B. (2019). Influence of the Organic Chain on the Optical Properties of Two-Dimensional Organic-Inorganic Hybrid Lead Iodide Perovskites. ACS Appl. Electron. Mater..

[72] Rossi D., Parobek D., Dong Y. (2017). Dynamics of Exciton-to-Mn Energy Transfer in Mn-Doped CsPbCl_3_ Perovskite Nanocrystals. J. Phys. Chem. C.

[73] Chen Y., Liu Y., Hong M. (2020). Cation-Doping Matters in Caesium Lead Halide Perovskite Nanocrystals: From Physicochemical Fundamentals to Optoelectronic Applications. Nanoscale.

[74] Yuan X., Ji S., De Siena M.C. (2017). Photoluminescence Temperature Dependence, Dynamics, and Quantum Efficiencies in Mn^2+^-Doped CsPbCl_3_ Perovskite Nanocrystals with Varied Dopant Concentration. Chem. Mater..

[75] Wei Q., Li M., Zhang Z. (2018). Efficient Recycling of Trapped Energies for Dual-Emission in Mn-Doped Perovskite Nanocrystals. Nano Energy.

[76] Parobek D., Roman B.J., Dong Y. (2016). Exciton-to-Dopant Energy Transfer in Mn-Doped Cesium Lead Halide Perovskite Nanocrystals. Nano Lett..

[77] Liu H., Wu Z., Shao J., Yao D., Gao H., Liu Y., Yu W., Zhang H., Yang B. (2017). CsPb_x_Mn_1−x_Cl_3_ Perovskite Quantum Dots with High Mn Substitution Ratio. ACS Nano.

[78] Labiadh H., Sellami B., Khazri A. (2017). Optical Properties and Toxicity of Undoped and Mn-Doped ZnS Semiconductor Nanoparticles Synthesized Through the Aqueous Route. Opt. Mater..

[79] Wu P., Yan X.P. (2013). Doped Quantum Dots for Chemo/Biosensing and Bioimaging. Chem. Soc. Rev..

[80] Lu X., Zhang J., Xie Y.N. (2018). Ratiometric Phosphorescent Probe for Thallium in Serum, Water, and Oil Samples Cased on Long-Lived, Spectrally Resolved, Mn-Doped ZnSe Quantum Dots and Carbon Dots. Anal. Chem..

[81] Diaz-Diestra D., Thapa B., Beltran-Huarac J. (2017). L-Cysteine Capped ZnS:Mn Quantum Dots for Room-Temperature Detection of Dopamine with High Sensitivity and Selectivity. Biosens. Bioelectron..

[82] Gong Y., Fan Z. (2014). Melamine-Modulated Mercaptopropionic Acid-Capped Manganese Doped Zinc Sulfide Quantum Dots as a Room-Temperature Phosphorescence Sensor for Detecting Clenbuterol in Biological Fluids. Sens. Actuators B-Chem..

[83] Tang D., Zhang J., Zhou R. (2018). Phosphorescent Inner Filter Effect-Based Sensing of Xanthine Oxidase and its Inhibitors with Mn-Doped ZnS Quantum Dots. Nanoscale.

[84] Zhang K., Yu T., Liu F. (2014). Selective Fluorescence Turn-On and Ratiometric Detection of Organophosphate Using Dual-Emitting Mn-Doped ZnS Nanocrystal Probe. Anal. Chem..

[85] Zhang C., Zhang K., Zhao T. (2017). Selective Phosphorescence Sensing of Pesticide Based on the Inhibition of Silver(I) Quenched ZnS:Mn^2+^ Quantum Dots. Sens. Actuators B-Chem..

[86] He H., Li C., Tian Y. (2016). Phosphorescent Differential Sensing of Physiological Phosphates with Lanthanide Ions-Modified Mn-Doped ZnCdS Quantum Dots. Anal. Chem..

[87] Zhang J., Tang D., Yao Y. (2018). Aggregation-Induced Phosphorescence Enhancement of Mn-Doped ZnS Quantum Dots: The Role of Dot-to-Dot Distance. Nanoscale.

[88] Liu W., Lin Q., Li H., Wu K., Robel I., Pietryga J.M., Klimov V.I. (2016). Mn^2+^-Doped Lead Halide Perovskite Nanocrystals with Dual-Color Emission Controlled by Halide Content. J. Am. Chem. Soc..

[89] De A., Mondal N., Samanta A. (2017). Luminescence Tuning and Exciton Dynamics of Mn-Doped CsPbCl_3_ Nanocrystals. Nanoscale.

[90] Li Z.-J., Hofman E., Davis A.H., Khammang A., Wright J.T., Dzikovski B., Meulenberg R.W., Zheng W. (2018). Complete Dopant Substitution by Spinodal Decomposition in Mn-Doped Two-Dimensional CsPbCl_3_ Nanoplatelets. Chem. Mater..

[91] Pradhan N. (2016). Red-Tuned Mn d-d Emission in Doped Semiconductor Nanocrystals. ChemPhysChem.

[92] Lin F., Li F., Lai Z. (2018). MnII-Doped Cesium Lead Chloride Perovskite Nanocrystals: Demonstration of Oxygen Sensing Capability Based on Luminescent Dopants and Host-Dopant Energy Transfer. ACS Appl. Mater. Interfaces.

[93] Li D., Li X., Zhao T. (2020). Ultraefficient Singlet Oxygen Generation from Manganese-Doped Cesium Lead Chloride Perovskite Quantum Dots. ACS Nano.

[94] Lin F.Y., Lai Z.W., Zhang L.C., Huang Y.P., Li F.M., Chen P.Y., Wang Y.R., Chen X. (2020). Fluorometric Sensing of Oxygen Using Manganese(II)-Doped Zinc Sulfide Nanocrystals. Microchim. Acta.

[95] Zhou Y., Chen J., Bakr O.M. (2018). Metal-Doped Lead Halide Perovskites: Synthesis, Properties, and Optoelectronic Applications. Chem. Mater..

[96] Jiang T., Ma W., Zhang H. (2021). Highly Efficient and Tunable Emission of Lead-Free Manganese Halides Toward White Light-Emitting Diode and X-Ray Scintillation Applications. Adv. Funct. Mater..

[97] Xu K., Meijerink A. (2018). Tuning Exciton-Mn^2+^ Energy Transfer in Mixed Halide Perovskite Nanocrystals. Chem. Mater..

[98] Bakthavatsalam R., Biswas A., Chakali M. (2019). Temperature-Dependent Photoluminescence and Energy-Transfer Dynamics in Mn^2+^-Doped (C_4_H_9_NH_3_)_2_PbBr_4_ Two-Dimensional 2D Layered Perovskite. J. Phys. Chem. C.

[99] Sun Q., Wang S., Zhao C. (2019). Excitation-Dependent Emission Color Tuning from an Individual Mn-Doped Perovskite Microcrystal. J. Am. Chem. Soc..

[100] Pinchetti V., Anand A., Akkerman Q.A. (2019). Trap-Mediated Two-Step Sensitization of Manganese Dopants in Perovskite Nanocrystals. ACS Energy Lett..

[101] Ricciarelli D., Meggiolaro D., Belanzoni P. (2021). Energy vs Charge Transfer in Manganese-Doped Lead Halide Perovskites. ACS Energy Lett..

[102] Brovelli S., Galland C., Viswanatha R. (2012). Tuning Radiative Recombination in Cu-Doped Nanocrystals Via Electrochemical Control of Surface Trapping. Nano Lett..

[103] Srivastava B.B., Jana S., Pradhan N. (2011). Doping Cu in Semiconductor Nanocrystals: Some Old and Some New Physical Insights. J. Am. Chem. Soc..

[104] Pradhan N., Sarma D.D. (2011). Advances in Light-Emitting Doped Semiconductor Nanocrystals. J. Phys. Chem. Lett..

[105] Pradhan N., Goorskey D., Thessing J. (2005). An Alternative of CdSe Nanocrystal Emitters: Pure and Tunable Impurity Emissions in ZnSe Nanocrystals. J. Am. Chem. Soc..

[106] Xie R., Peng X. (2009). Synthesis of Cu-Doped InP Nanocrystals (d-dots) with ZnSe Diffusion Barrier as Efficient and Color-Tunable NIR Emitters. J. Am. Chem. Soc..

[107] Sarkar S., Karan N.S., Pradhan N. (2011). Ultrasmall Color-Tunable Copper-Doped Ternary Semiconductor Nanocrystal Emitters. Angew. Chem. Int. Ed..

[108] Zhang W., Zhou X., Zhong X. (2012). One-Pot Noninjection Synthesis of Cu-Doped Zn_x_Cd_1−x_S Nanocrystals with Emission Color Tunable Over Entire Visible Spectrum. Inorg. Chem..

[109] Sheng X., Liu Y., Wang Y. (2017). Cesium Lead Halide Perovskite Quantum Dots as a Photoluminescence Probe for Metal Ions. Adv. Mater..

[110] Liu Y., Tang X., Zhu T. (2018). All-Inorganic CsPbBr_3_ Perovskite Quantum Dots as a Photoluminescent Probe for Ultrasensitive Cu^2+^ Detection. J. Mater. Chem. C.

[111] Zhang L., Yue S., Li B. (2012). A Series of [Cu(N–N)(P–P)]B_F4_ Complexes: Luminescence Quenching Caused by Electron-Configuration Transformation in Excited State. Inorg. Chim. Acta.

[112] Shi L., Li B., Yue S. (2009). Synthesis, Photophysical and Oxygen-Sensing Properties of a Novel Bluish-Green Emission Cu(I) Complex. Sens. Actuators B-Chem..

[113] Zhang L.C., Lin F.Y., Ye M., Tian D.J., Jin J.W., Huang Y.P., Jiang Y.Q., Wang Y.R., Chen X. (2021). Luminescence Sensing of Oxygen Using Copper Iodide Hybrid Material. Sens. Actuators B-Chem..

[114] Lan T., Wang W. (2018). Electrospinning Fibrous Films Doped with a Series of Luminescent Copper Complexes: Synthesis, Characterization and Oxygen Sensing Performance Comparison. Sens. Actuators B-Chem..

[115] Huitorel B., El Moll H., Utrera-Melero R. (2018). Evaluation of Ligands Effect on the Photophysical Properties of Copper Iodide Clusters. Inorg. Chem..

[116] Xie M., Han C., Liang Q., Zhang J., Xie G., Xu H. (2019). Highly Efficient Sky Blue Electroluminescence from Ligand-Activated Copper Iodide Clusters: Overcoming the Limitations of Cluster Light-Emitting Diodes. Sci. Adv..

[117] Yang L.O., Zhang L.C., Tian D.J., Ye M., Xi C. (2023). Oxygen Sensing Behavior Modulation of Tetranuclear Copper Iodide Hybrid Materials Using Ligand Engineering. Sens. Actuators B-Chem..

[118] Yang L.O., Tian D.J., Liu X.L., Lin F.Y., Xi C. (2024). Oxygen Sensing Performance Modulation Using Luminescent Ionic Copper Iodide Clusters Based on Mn(II) Crystal Field Intensity. Sens. Actuators B-Chem..

[119] Zhao Y.M., Geng X., Shi X.X., Guo Y., Sun Y., Qu L., Li Z. (2021). A Fluorescence-Switchable Carbon Dot for the Reversible Turn-On Sensing of Molecular Oxygen. J. Mater. Chem. C.

[120] He Z., Wang Y.N., An J.H. (2024). Splitting and Aggregation of Carbon Dots: Wavelength-Shifted and Ratiometric Fluorescence Sensing of Peroxynitrite. Anal. Chem..

